# High Intrapulmonary Rifampicin and Isoniazid Concentrations Are Associated With Rapid Sputum Bacillary Clearance in Patients With Pulmonary Tuberculosis^[Author-notes ciac228-FM1]^

**DOI:** 10.1093/cid/ciac228

**Published:** 2022-03-23

**Authors:** Andrew D McCallum, Henry E Pertinez, Aaron P Chirambo, Irene Sheha, Madalitso Chasweka, Rose Malamba, Doris Shani, Alex Chitani, Jane E Mallewa, Jamilah Z Meghji, Jehan F Ghany, Elizabeth L Corbett, Stephen B Gordon, Geraint R Davies, Saye H Khoo, Derek J Sloan, Henry C Mwandumba

**Affiliations:** Department of Clinical Sciences, Liverpool School of Tropical Medicine, Liverpool, United Kingdom; Malawi-Liverpool-Wellcome Clinical Research Programme, Kamuzu University of Health Sciences, Blantyre, Malawi; Department of Pharmacology, University of Liverpool, Liverpool, United Kingdom; Department of Pharmacology, University of Liverpool, Liverpool, United Kingdom; Malawi-Liverpool-Wellcome Clinical Research Programme, Kamuzu University of Health Sciences, Blantyre, Malawi; Malawi-Liverpool-Wellcome Clinical Research Programme, Kamuzu University of Health Sciences, Blantyre, Malawi; Malawi-Liverpool-Wellcome Clinical Research Programme, Kamuzu University of Health Sciences, Blantyre, Malawi; Malawi-Liverpool-Wellcome Clinical Research Programme, Kamuzu University of Health Sciences, Blantyre, Malawi; Malawi-Liverpool-Wellcome Clinical Research Programme, Kamuzu University of Health Sciences, Blantyre, Malawi; Department of Medicine, Kamuzu University of Health Sciences, Blantyre, Malawi; Department of Medicine, Kamuzu University of Health Sciences, Blantyre, Malawi; Department of Clinical Sciences, Liverpool School of Tropical Medicine, Liverpool, United Kingdom; Malawi-Liverpool-Wellcome Clinical Research Programme, Kamuzu University of Health Sciences, Blantyre, Malawi; Department of Radiology, Royal Liverpool and Broadgreen University Hospitals, Liverpool, United Kingdom; Department of Clinical Research, London School of Hygiene and Tropical Medicine, London, United Kingdom; Department of Clinical Sciences, Liverpool School of Tropical Medicine, Liverpool, United Kingdom; Malawi-Liverpool-Wellcome Clinical Research Programme, Kamuzu University of Health Sciences, Blantyre, Malawi; Institute of Infection, Veterinary and Ecological Sciences, University of Liverpool, Liverpool, United Kingdom; Department of Pharmacology, University of Liverpool, Liverpool, United Kingdom; Infection and Global Health Division, University of St Andrews, St Andrews, United Kingdom; Department of Clinical Sciences, Liverpool School of Tropical Medicine, Liverpool, United Kingdom; Malawi-Liverpool-Wellcome Clinical Research Programme, Kamuzu University of Health Sciences, Blantyre, Malawi

**Keywords:** tuberculosis, pharmacokinetics, pharmacodynamics, antibiotics, antitubercular

## Abstract

**Background:**

Intrapulmonary pharmacokinetics may better explain response to tuberculosis (TB) treatment than plasma pharmacokinetics. We explored these relationships by modeling bacillary clearance in sputum in adult patients on first-line treatment in Malawi.

**Methods:**

Bacillary elimination rates (BER) were estimated using linear mixed-effects modelling of serial time-to-positivity in mycobacterial growth indicator tubes for sputum collected during the intensive phase of treatment (weeks 0–8) for microbiologically confirmed TB. Population pharmacokinetic models used plasma and intrapulmonary drug levels at 8 and 16 weeks. Pharmacokinetic-pharmacodynamic relationships were investigated using individual-level measures of drug exposure (area-under-the-concentration-time-curve [AUC] and *C*_max_) for rifampicin, isoniazid, pyrazinamide, and ethambutol, in plasma, epithelial lining fluid, and alveolar cells as covariates in the bacillary elimination models.

**Results:**

Among 157 participants (58% human immunodeficiency virus [HIV] coinfected), drug exposure in plasma or alveolar cells was not associated with sputum bacillary clearance. Higher peak concentrations (*C*_max_) or exposure (AUC) to rifampicin or isoniazid in epithelial lining fluid was associated with more rapid bacillary elimination and shorter time to sputum negativity. More extensive disease on baseline chest radiograph was associated with slower bacillary elimination. Clinical outcome was captured in 133 participants, with 15 (11%) unfavorable outcomes recorded (recurrent TB, failed treatment, or death). No relationship between BER and late clinical outcome was identified.

**Conclusions:**

Greater intrapulmonary drug exposure to rifampicin or isoniazid in the epithelial lining fluid was associated with more rapid bacillary clearance. Higher doses of rifampicin and isoniazid may result in sustained high intrapulmonary drug exposure, rapid bacillary clearance, shorter treatment duration and better treatment outcomes.

## Teaser

First-line anti-tuberculosis medicines exhibit variable pharmacokinetics. Modeling of serial sputum time-to-positivity data enables estimation of sputum bacillary elimination. Increased intrapulmonary rifampicin or isoniazid exposure (*C*_max_or area-under-the-concentration-time-curve [AUC]) was associated with rapid bacillary clearance in pulmonary tuberculosis.

## INTRODUCTION

The pharmacokinetics (PK) of first-line tuberculosis (TB) drugs are highly variable, with suboptimal drug exposure inconsistently linked to poor treatment outcomes [1]. Although most studies have focused on drug concentrations in peripheral blood, few have appraised drug exposure-response relationships at, or near to, the pulmonary site of infection. We have recently described the intrapulmonary PK for rifampicin, isoniazid, pyrazinamide, and ethambutol in a cohort of Malawian adults with pulmonary TB [2], demonstrating that intrapulmonary concentrations differ substantially from plasma and that rifampicin concentrations were low in all compartments.

Rifamycins are central to anti-tuberculosis treatment through their ability to sterilize lesions and provide relapse-free cure [3]. Preclinical models and phase 2 trials have demonstrated a relationship between rifamycin exposure and reduction in bacillary burden [4–6], while a recent phase 3 clinical trial of a 4-month regimen containing high-dose rifapentine with a fluoroquinolone was noninferior to the standard 6-month regimen [7]. We postulated that higher intrapulmonary concentrations, particularly for rifampicin, may be associated with greater bacillary clearance.

Serial quantitative bacteriology can be used to chart the decline in *Mycobacterium tuberculosis* bacillary load over time on treatment [8]. The time-to-positivity (TTP) after inoculation of processed sputum into liquid culture provides an inverse measure of bacillary load [9]. Studies extending out for 8–12 weeks may deploy statistical modeling techniques to summarize evolving patterns of bacillary clearance over a longer period [10], and correlation has been described between bacillary clearance rates and long-term TB outcomes [9].

Earlier regression analysis of our pharmacokinetic-pharmacodynamic (PK-PD) dataset did not show any relationship between plasma or intrapulmonary drug exposure, and the endpoints of 2-month sputum culture conversion or relapse-free cure to 18 months [2], but suggested a relationship between maximum concentration (*C*_max_) of rifampicin and isoniazid in epithelial lining fluid and favorable outcome (odds ratio [OR] 2.03 [0.97–9.24], *P* = .221 and OR 3.79 [0.96–17.86], *P* = .071, respectively). In this study, we explore the relationship between plasma and intrapulmonary drug exposure and sputum bacillary clearance, with a view to identifying potential targets for therapeutic interventions to shorten or optimize TB treatment.

## METHODS

### Study Participants

A prospective cohort study was conducted at Queen Elizabeth Central Hospital in Blantyre, Malawi, between 2016 and 2018. Details of recruitment, and inclusion and exclusion criteria have been described previously [2]. In brief, consenting adults aged 16–65 years with sputum smear- or Xpert MTB/RIF-positive, drug-sensitive, pulmonary TB on National Tuberculosis Control Programme diagnostic samples were eligible for enrollment. The median age of the cohort was 34 years (interquartile range [IQR]: 28–39), and 76% (120/157) were male. Fifty-eight percent (91/157) were infected with human immunodeficiency virus (HIV).

Participants received daily fixed-dose combination tablets containing rifampicin, isoniazid, pyrazinamide, and ethambutol for 8 weeks, followed by 16 weeks of rifampicin and isoniazid according to a World Health Organization (WHO)-approved weight-adjusted regimen and national guidelines [11]. All patients had HIV antibody testing, and those testing positive received antiretroviral therapy according to national guidelines [12]. Baseline chest radiographs (CXR) were scored independently by 2 readers using a published method [13]. In brief, the total percentage of lung affected by any pathology was estimated (score 0–100), and an additional 40 points added for cavitation [13]. The total CXR score ranged between 0 and 140, and the top 5% most discordant films were re-read and scored by consensus. Participants were followed up over the course of TB treatment and for 1 year after end-of-treatment.

### PK Sampling and Analysis

Details of PK sampling and modelling have been described previously [2]. Briefly, participants returned for 2 PK sampling visits at 7–8 weeks and 15–16 weeks from start of TB treatment. All participants contributed plasma for pharmacokinetic sampling. At enrollment into the cohort, every third participant was selected to also contribute intrapulmonary samples. Those in the intrapulmonary arm had a research bronchoscopy and bronchoalveolar lavage to collect samples of epithelial lining fluid and alveolar cells, coinciding with their plasma PK sampling visits on weeks 7–8 and 15–16. Drug concentrations in plasma, epithelial lining fluid, and alveolar cells were determined using liquid chromatography/tandem mass spectrometry.

Population PK models were developed using NONMEM^©^ (version 7.4.0, ICON Development Solutions). The final population PK models described the plasma and intrapulmonary PK for each drug and were used to generate individual-level post hoc Bayesian estimates of plasma and intrapulmonary area-under-the-concentration-time-curve (AUC) and *C*_max_.

### Sputum Sample Collection, Processing, and Bacteriology

Participants were allocated sequentially to staggered, balanced sampling blocks. Sputum samples were collected from participants allocated to Block 1 on days 0, 7, 21, 35, and 49 of TB treatment, and from participants allocated to Block 2 on days 0, 14, 28, 42, and 56 of TB treatment. Twelve-hour overnight sputum samples were collected [14].

All participants submitted spot sputum samples at end-of-treatment to assess bacteriological cure. Those with ongoing or recurrent symptoms submitted post-treatment samples to assess for recurrent disease but were not used in bacillary elimination rate (BER) analysis. All sputum samples were processed in the TB Laboratory at the Kamuzu University of Health Sciences. The laboratory participated in quality control through the UK National External Quality Assessment Programme.

Sputum samples were processed for smear microscopy [15] and liquid culture within 24 hours. Baseline samples were also processed on the Xpert MTB/RIF assay (Cepheid) to confirm rifampicin sensitivity. Sputum samples (1 ml) were decontaminated with *N*-acetyl-L-cysteine/sodium hydroxide (NALC-NaOH) 3% and inoculated into mycobacteria growth indicator tubes (MGIT, Becton Dickinson). TTP (in hours), recorded by the Automated Mycobacterial Detection System, was used as an inverse measure of bacillary load. After a maximum of 42 days, the instrument flags the tube as negative if no growth has been detected. Ziehl-Neelsen microscopy, examination for cording, and TBc Identification Test kits (Becton Dickinson) were used to confirm that positive isolates represented growth of *Mycobacterium tuberculosis*. Positive isolates were incubated on blood agar to exclude mixed contamination of samples.

Baseline drug susceptibility of screening isolates was measured on custom-made microtiter plates (UKMYC3 Sensititer, Thermo Scientific) [2]. These assays used 96-well plates with doubling concentrations of rifampicin (0.015–16 µg/mL), isoniazid (0.015–16 µg/mL), and ethambutol (0.25–16 µg/mL). Pyrazinamide was not assessed due to its’ need for acidic test conditions. The minimum inhibitory concentration (MIC) was recorded as the lowest concentration with no visible growth for each antibiotic.

### Data Analysis and Statistical Methods

BER modeling employed nonlinear mixed effects methods [9, 10]. Participants with ≥ 2 TTP measurements were included. TTP results above the limit of quantification in “negative” sputum samples (42 days using the MGIT 960) were accounted for using a partial likelihood method [16, 17]. TTP data from sputum samples out to day 56 of treatment (end of the intensive phase) were used in the BER modeling.

TTP data were transformed into the log of the reciprocal (log_10_(1/TTP)) to show decline over time. Attempts to model curvature with quadratic and spline functions did not converge, and consequently a linear mixed effects model was adopted. This took the form:


$$\begin{array}{l} log_{10}\left(\frac{1}{TTP}\right)=a+b*daysontreatment, \end{array}$$

where *a* represented the intercept (modeled baseline bacillary load), and *b* the slope (BER). Random effects on *a* and *b* were included in the model to capture inter-individual variability.

Log-transformed PK indices (AUC, *C*_max_, AUC/MIC, and *C*_max_/MIC) were included as covariates in the BER model. Covariates were added to the base model as shown below:


$$\begin{array}{l} BER=\theta_{BER}+\left(\theta_{PK}*\left(\frac{PK}{mean_{PK}}\right)\right). \end{array}$$

The typical value for BER was calculated from the sum of the fixed effect/population estimate for BER (θ_BER_), and the fixed effect for the log-transformed PK parameter (θ_PK_) multiplied by the standardized PK parameter. Inter-individual effects were included on BER. Model selection was achieved using likelihood ratio testing with the minimum objective function value (OFV) as the criterion. PK-BER relationships were considered significant if associated with a decrease in OFV of >3.84 (*P* = .05, χ^2^ distribution, 1 degree of freedom), with improved goodness-of-fit plots and parameter estimate precision.

Model building steps and associated data analysis were managed using the software utilities Pirana (version 2.9.6), Xpose (version 4.6.1), and *R* (version 3.5.0) [18].

### Ethical Approval

Ethical approval was obtained from the Research Ethics Committees of the College of Medicine, University of Malawi (P.09/15/1800), and the Liverpool School of Tropical Medicine (15.033).

## RESULTS

### Participants and Samples

As detailed in Supplementary Figure 1, 157 Malawian adults with pulmonary TB were recruited for follow up over 18 months [2]. Of the eligible participants, 136 (87%) remained in the study until the end of the intensive phase of treatment (week 8), 130 (83%) until the end of treatment (week 24), and 125 (80%) completed 18 months of follow-up.

Nine-hundred-and-seventy-two sputum samples were collected and processed in liquid culture. Of these, 13% (122/972) of samples were contaminated and removed from further TTP analysis. One-hundred-and-forty-two participants (90.4%) had at least 1 uncontaminated sputum culture result to enable assessment of bacteriological endpoints. Mean baseline TTP was 9.5 days (standard deviation 8.8 days), and most participants (98/157, 69%) were 2+ or 3+ for acid-fast bacilli on baseline smear microscopy.

### Baseline Drug Sensitivity

No participants had rifampicin-resistance detected (MIC breakpoint 1 µg/mL) [19]. Six (6.8%) participants had isoniazid MICs above breakpoints for resistance (0.2 µg/mL), and 1 further participant had MICs above breakpoints for both isoniazid and ethambutol (5 µg/mL). Four (4.6%) participants had ethambutol MICs above 5 µg/mL. There was no significant relationship between isoniazid resistance and BER (*P* = .674), 2-month culture conversion (*P* = .642), or final outcome (*P* = 1.000).

### Evaluation of Bacillary Elimination

Five hundred and eleven TTP results, from 125 participants, were included in the model. CXR score was closely associated with baseline bacillary load and was included as a covariate on the intercept to adjust for baseline (radiological) severity of disease. This was associated with a drop in objective function of −5.536 (*P* < .05) and improved parameter estimates. The final model took the form:


$$\begin{array}{l} log_{10}\left(\frac{1}{TTP}\right)=\left(a+\left(\frac{CXR}{33.4}\right)\right)+b*daysontreatment, \end{array}$$

where CXR score was normalized to the population mean score of 33.4. Exponential interindividual variability was supported on slope (*a*) and CXR score, and on bacillary elimination rate (*b*).

Supplementary Table 1 describes the final parameter estimates from the BER model. The model estimated a baseline TTP of 9.5 days (transformed TTP −.979), with an increase in TTP of 1.04 per day on treatment (transformed TTP −.016).

### Predictors of Bacillary Elimination Rate

**Figure 1.**
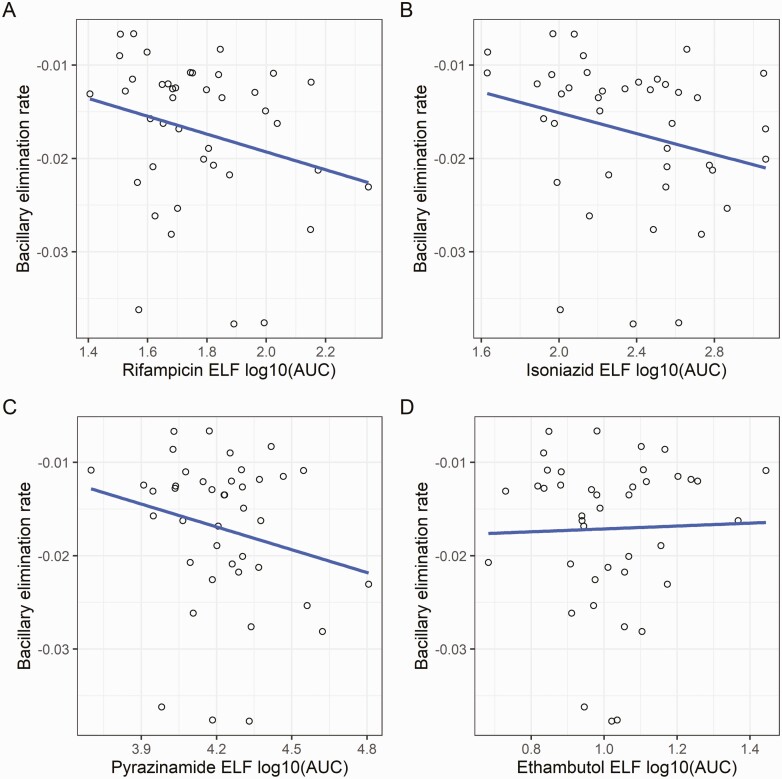
Epithelial lining concentrations and bacillary elimination rate. Higher rifampicin (*A*) and isoniazid (*B*) ELF AUC were associated with more rapid sputum bacillary elimination (more negative BER) when added to the PK-PD model (Δ objective function value ≥3.84, *P* = .05, χ^2^ distribution, 1 degree of freedom). Line of best fit shown for illustration. PK data have been log-transformed. Abbreviations: AUC, area under the concentration-time curve; BER, bacillary elimination rate; ELF, epithelial lining fluid.

BER increased with greater exposure to rifampicin (*C*_max_ or AUC) and isoniazid in the epithelial lining fluid (*C*_max_ or AUC; ΔOFV −5.009, −6.014−4.741, and −5.248, respectively, Table 1, Figure 1). Drug concentrations in plasma or alveolar cells were not significantly associated with sputum bacillary elimination rates. Inclusion of MIC values into the PK-PD model was not associated with improvement in model fit.

**Table 1.** Pharmacokinetic Parameters and Sputum Bacillary Elimination Rates

Drug	Matrix	Model Fit: No MIC Data				Model Fit: Including MIC Data			
		PK Index	n	ΔBER	ΔOFV	PK Index	n	ΔBER	ΔOFV
Rifampicin	Plasma	AUC	125	0.0002	0.000	AUC/MIC	70	0.0258	–2.047
		*C*_max_	125	0.0078	–1.954	*C*_max_/MIC	70	0.0246	–2.002
	Epithelial lining fluid	AUC	42	–0.0035	**–6.014**	AUC/MIC	30	–0.0035	–2.808
		*C*_max_	42	–0.0020	**–5.009**	*C*_max_/MIC	30	–0.0020	–1.069
	Alveolar cells	AUC	42	0.0012	–0.070	AUC/MIC	30	0.0012	–0.921
		*C*_max_	42	0.0010	–0.003	*C*_max_/MIC	30	0.0010	–1.710
Isoniazid	Plasma	AUC	125	–0.0035	–0.166	AUC/MIC	70	–0.0014	–0.283
		*C*_max_	125	0.0019	–0.112	*C*_max_/MIC	70	–0.0008	–0.159
	Epithelial lining fluid	AUC	42	–0.0262	**–5.248**	AUC/MIC	30	–0.0088	–2.646
		*C*_max_	42	–0.0145	**–4.741**	C_max_/MIC	30	–0.0085	–2.120
	Alveolar cells	AUC	42	–0.0027	–0.210	AUC/MIC	30	0.0023	–0.149
		*C*_max_	42	–0.0001	–0.001	*C*_max_/MIC	30	0.0030	–0.332
Pyrazinamide	Plasma	AUC	125	–0.0237	–0.816	…	…	…	…
		*C*_max_	125	–0.0026	–0.017	…	…	…	…
	Epithelial lining fluid	AUC	42	–0.0586	–2.544	…	…	…	…
		*C*_max_	42	–0.0310	–2.289	…	…	…	…
	Alveolar cells	AUC	42	0.0219	–0.681	…	…	…	…
		*C*_max_	42	0.0121	–1.242	…	…	…	…
Ethambutol	Plasma	AUC	125	–0.0048	–2.301	AUC/MIC	70	–0.0021	–1.857
		*C*_max_	125	–0.0002	–0.052	*C*_max_/MIC	70	–0.0002	–0.010
	Epithelial lining fluid	AUC	42	0.0008	–0.005	AUC/MIC	30	–0.0063	–2.041
		*C*_max_	42	0.0008	–0.013	*C*_max_/MIC	30	–0.0028	–1.511
	Alveolar cells	AUC	42	0.0162	–1.063	AUC/MIC	30	0.0024	–0.092
		*C*_max_	42	0.0119	–0.804	*C*_max_/MIC	30	0.0004	–0.007

Pharmacokinetic parameters added to base bacillary elimination rate (BER) model as a covariate on slope. Analysis restricted to those with observations in the matrix/ compartment of interest. MIC data not available for pyrazinamide. ΔBER shows the effect of the pharmacokinetic parameter on the bacillary elimination rate, with negative values reflecting faster elimination. Models adjusted for baseline extent of disease by including chest radiograph score as a covariate on intercept. Pharmacokinetic-BER relationships were considered significant if associated with a decrease in objective function value of >3.84 (ΔOFV, *P* = .05, χ^2^ distribution, one degree of freedom), as shown in bold.

Abbreviations: AUC, area under the concentration time curve; BER, bacillary elimination rate; C_max_, maximal concentration; MIC, minimum inhibitory concentration; OFV, objective function value; PK, pharmacokinetics.

Chest radiograph score was strongly associated with baseline bacillary load and included in the base model. No correlation was seen between the radiological extent of right midzone disease and intrapulmonary drug concentration [2]. Clinical predictors of BER were explored in univariate and multivariate analysis (Table 2). Longer symptom duration, HIV coinfection, higher baseline white cell count, higher creatinine clearance, and greater CXR score were associated with slower bacillary elimination. On multivariate analysis, higher baseline pulse, respiratory rate and ALT were associated with more rapid BER.

**Table 2.** Clinical Predictors of Bacillary Elimination Rate

Characteristic	Total (n = 125)^a^	Univariate Analysis		Multivariate Analysis	
		Effect on BER (SE)^b^	*P* value	Effect on BER (SE)^b^	*P* Value
Age (years), median [IQR]	34 [28–39]	−0.038 × 10^-3^ (−0.074 × 10^-3^)	0.608	…	…
Male sex, n (%)	95 (76)	−0.959 × 10^-3^ (−0.629 × 10^-3^)	0.530	…	…
Duration of symptoms in weeks, median [IQR]	4 (3–8)	0.305 × 10^-3^ (0.158 × 10^-3^)	0.056	0.271 × 10^-3^ (0.151 × 10^-3^)	.075
Missed a total of > 2 doses of TB treatment, n (%)	7 (6)	−0.127 × 10^-3^ (2.84 × 10^-3^)	0.964	…	…
HIV-infected, n (%)	68 (54)	−2.510 × 10^-3^ (1.290 × 10^-3^)	0.054	0.575 × 10^-3^ (1.320 × 10^-3^)	.664
Baseline CD4 in cells/mm^3^, median [IQR]	312 [160–464]	0.005 × 10^-3^ (0.003 × 10^-3^)	0.079 ^c^	…	…
Baseline body mass index in kg/m^2^, median [IQR]	18 [17–20]	0.112 × 10^-3^ (0.287 × 10^-3^)	0.697	…	…
Change in weight over treatment in kg, median [IQR]	5 [3–8]	0.159 × 10^-3^ (0.157 × 10^-3^)	0.314	…	…
Baseline pulse in beats/min, median [IQR]	103 [92–115]	−0.108 × 10^-3^ (0.032 × 10^-3^)	0.001	−0.098 × 10^-3^ (0.033 × 10^-3^)	.003
Baseline respiratory rate in breaths/min, median [IQR]	21 [17–24]	−0.297 × 10^-3^ (0.152 × 10^-3^)	0.052	−0.291 × 10^-3^ (0.145 × 10^-3^)	.048
Baseline hemoglobin in g/dL, median [IQR]	11 [10–12]	0.271 × 10^-3^ (0.350 × 10^-3^)	0.441	…	…
Baseline white cell count (×10^3^/μL), median [IQR]	7 [6–9]	0.411 × 10^-3^ (0.244 × 10^-3^)	0.094	0.406 × 10^-3^ (0.249 × 10^-3^)	.106
Baseline creatinine clearance in mL/min, median [IQR]	110 [86–132]	0.031 × 10^-3^ (0.019 × 10^-3^)	0.099	0.032 × 10^-3^ (0.018 × 10^-3^)	.079
Baseline ALT in IU/L, median [IQR]	19 [13–30]	−0.103 × 10^-3^ (0.037 × 10^-3^)	0.006	−0.078 × 10^-3^ (0.036 × 10^-3^)	.031
CXR score, median [IQR]	22 [8–60]	0.057 × 10^-3^ (0.023 × 10^-3^)	0.012	0.028 × 10^-3^ (0.024 × 10^-3^)	.242
Presence of cavities on CXR, n (%)	49 (39)	3.240 × 10^-3^ (1.130 × 10^-3^)	0.015	…	…

Abbreviations: ALT, alanine transaminase; BER, bacillary elimination rate; CXR, chest radiograph; HIV, human immunodeficiency virus; IQR, interquartile range; SE, standard error; TB, tuberculosis.

Continuous variables presented as median [IQR], categorical variables as total (%).

Effect on bacillary elimination rate (BER) shows the change in BER associated with a 1-unit change in the predictor variable, multiplied by x10^3^. Negative estimates are associated with more rapid bacillary clearance.

Not included in multivariate model due to significant correlation with HIV status.

### Culture Conversion and Clinical Outcomes

**Figure 2.**
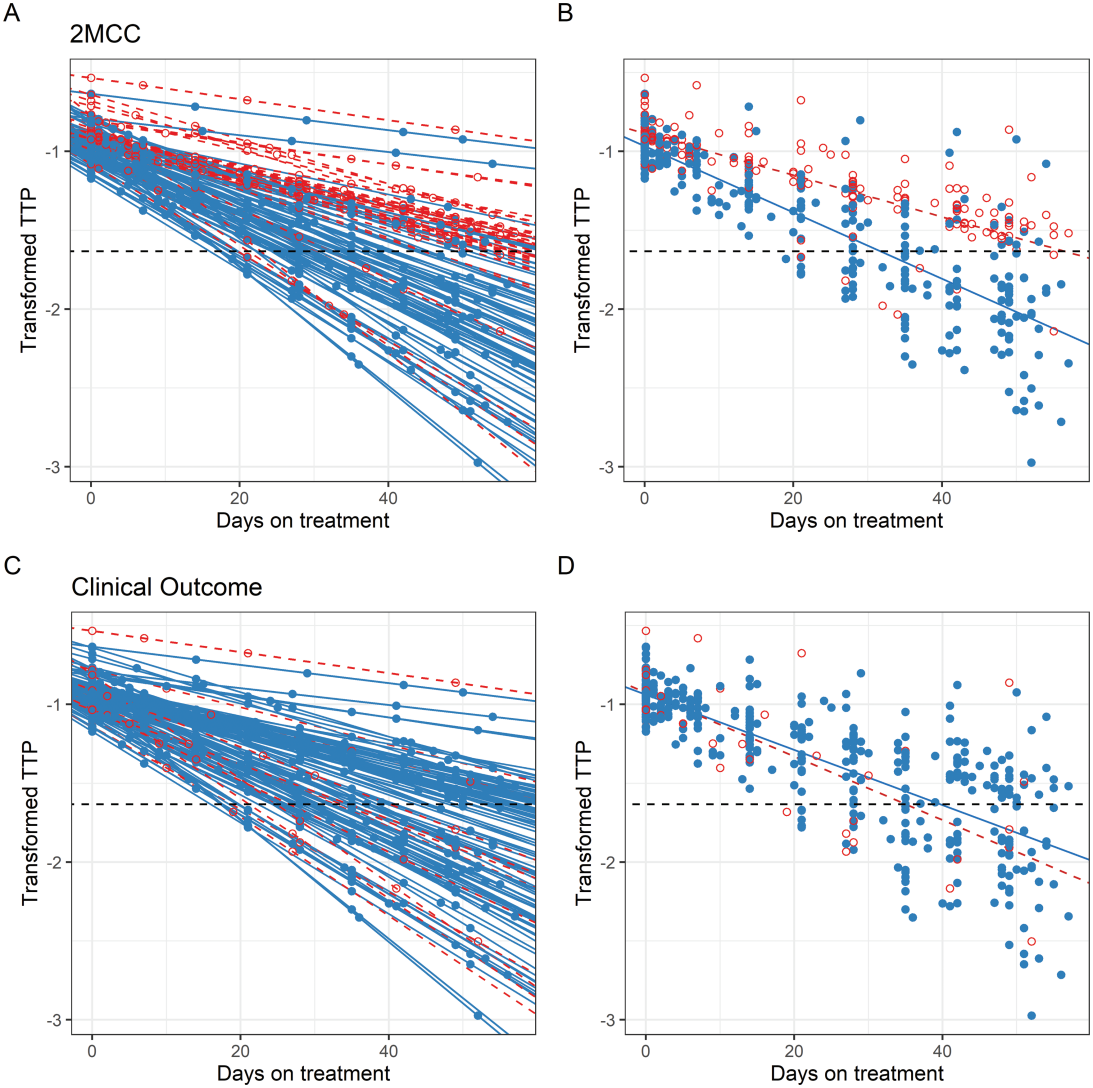
Modelled bacillary elimination rate and time on treatment, stratified by 2-month culture conversion and clinical outcome. Serial TTP data from each participant included in the model (n = 125), with each line representing an individual participant and each point the modeled transformed TTP (log_10_(1/TTP)) as a measure of bacillary load. The rate of reduction in transformed TTP represents the sputum bacillary elimination rate. The dashed horizonal line is the limit of quantification for MGIT data at −1.623 (log_10_(1/42 days)), with partial likelihood modeling [16, 17] accounting for samples beyond the limit of quantification. In the top panels (*A*, *B*), solid lines and points represent samples from those participants that culture converted by 2 months (2MCC), dashed lines and hollow points those participants that failed to culture convert.  *A*, Each line represents an individual participant. *B*, The lines summarize the bacillary elimination rate for sputum culture converters and nonconverters. In the bottom panels (*C*, *D*), the solid lines and points represent those participants with favorable clinical outcomes, the dashed lines and hollow points those with unfavorable clinical outcomes. The dashed black horizonal line is the lower limit of quantification for MGIT TTP data at −1.623 (log_10_(1/42 days)). Abbreviations: MGIT, mycobacteria growth indicator tube; TTP, time to positivity. C, Each line represents an individual participant. D, The lines summarize the bacillary elimination rate for those with favorable and unfavorable outcomes.

One hundred and twenty-six participants had sufficient data to assess 2-month culture conversion: 81 (64%) had stable culture conversion by 2 months. BER was closely correlated with rates of culture conversion (Figure 2). The median modelled time-to-culture conversion was 32 days (IQR: 25–41) in those that had culture-converted by 2 months, versus 61 days (IQR: 54–69) in those that had not. Clinical outcome was captured in 133 participants, with 15 (11%) unfavorable outcomes recorded: 9 treatment failures, 6 recurrent TB disease (Supplementary Figure 1).  No relationship between BER and late clinical outcome was identified (Figure 2).

## DISCUSSION

This novel study describes the intra- and extracellular PK-PD of first-line anti-TB drugs at the pulmonary site of infection in a cohort of patients with TB. We demonstrate that increased exposure (*C*_max_ or AUC) to rifampicin and isoniazid in epithelial lining fluid was associated with more rapid bacillary clearance from the sputum. By contrast, drug exposure in plasma or alveolar cells was not associated with response to treatment, by either sputum bacillary elimination rates, 2-month culture conversion, or late clinical outcome [2]. Higher doses of rifampicin and isoniazid may result in sustained high intrapulmonary drug exposure and rapid bacillary clearance, with potential to shorten treatment, interrupt transmission, and improve treatment outcomes.

Accumulating evidence suggests that high-dose rifamycin use is associated with improved bacillary clearance rates [4–6, 20–22] and offers treatment shortening potential when combined with fluoroquinolones [7]. Even at a fixed 10 mg/kg rifampicin dose, there was sufficient inter-individual PK variability for us to document more rapid bacillary clearance from sputum in those patients with higher intrapulmonary drug concentrations. Given that that 10 mg/kg lies at the bottom of a dose-exposure-response curve [6], and increased rifampicin doses are associated with “super-proportional” increases in plasma antibiotic exposure [21], dose increases will likely increase bacillary clearance and may improve outcomes. Indeed, quadrupling doses to 40 mg/kg have recently been reported as well-tolerated [23].

Previous studies have linked plasma isoniazid concentrations to rates of 2-month culture conversion [24, 25]. Here we observed that those patients with higher AUC or *C*_max_ in epithelial lining fluid achieved more rapid bacillary clearance. Isoniazid is thought to act on extracellular bacteria in log phase growth [8], and those patients with high isoniazid drug concentrations in epithelial lining fluid may achieve more rapid bacillary elimination in sputum through early reduction in this bacillary subpopulation.

Bacillary elimination rates have shown promise as a surrogate for long-term response [9]. As a continuous measure, BER provides more information on the antibacterial effect of therapy over the entire study period than a binary measurement at a single time point. Regimens that optimise rates of bacillary elimination from sputum may have an important role in reducing transmissibility [26]. Furthermore, as higher bacillary loads have been associated with more tissue destruction [27], more rapid reduction in bacillary load may limit further destruction and reduce long-term morbidity of post-TB lung disease.

It was possible to generate the BER for most participants in this cohort. We adopted novel design features, including staggered sputum sampling and overnight sputum samples to maximize information for BER PD modeling. Staggered sampling allowed for sample collection over a greater number of time points, whereas overnight samples increased the microbiological yield and enabled more positive culture results to inform the PK-PD modelling. Addressing missingness in TTP data set—due to reversion to culture negativity, failure to expectorate, or contamination—is challenging but was addressed by adoption of a partial likelihood approach [16, 17]. This enabled the construction of a PD model with random effects on both intercept and slope, incorporating data above the limit of detection of 42 days. This had the benefit of allowing for the estimation of BER for more participants and removing bias induced by restricting analysis to only positive samples.

Alongside rifampicin and isoniazid drug exposure, radiological extent of disease was closely associated with BER. The CXR score captured the extent of parenchymal disease and the presence or absence of cavitation. The relationship between bacillary load and CXR cavitation is well recognized [26, 28, 29], with resected cavities containing as many as 10^7^-10^9^ organisms/cavity as compared to 10^2^–10^4^/caseum in caseous necrosis [30]. Interestingly, these data suggest that those with more severe disease on baseline CXR have slower bacillary elimination rates: potentially secondary to anatomical disruption, greater initial bacterial burden, or loss of immunological control of infection, and emphasizes the importance of early diagnosis and rapid entry to treatment. Currently, ATS guidelines recommend prolongation of treatment for those microbiologically-confirmed cavitary disease [31].

BER was not associated with late clinical outcomes in this cohort. This may be explained by the high treatment success rate with few unfavorable outcomes and also by potential for exogenous reinfection. Without resources to genotype cases of recurrent TB, reinfection with new strains of *M. tuberculosis* cannot be distinguished from relapse by the original infecting strain in late clinical outcomes, although early recurrent disease is known to more likely represent relapse than reinfection in other cohorts [32]. Regardless of association with late clinical outcomes, strategies that increase bacillary elimination rates in sputum, such as higher dosing to optimise intrapulmonary PK, may limit further tissue damage, reduce the period of infectiousness, and are of public health importance.

Alveolar macrophages represent the main reservoir of intracellular mycobacteria [33] and may be postulated to represent a pharmacological sanctuary. It was an interesting observation in this study that alveolar cell drug exposure was not associated with any marker of response. Prior work has suggested that rifampicin may distribute reasonably well into lung lesions [34, 35]. Accumulation of drug within macrophages in necrotic foci may be key to sterilization of caseum, and this effect would not necessarily be captured by assessment of bacillary clearance in sputum. Our results may also be explained by technical challenges involved in measuring PK in this compartment, including measurement of cell-associated drug or subcellular partitioning of drug and mycobacteria [35], limiting our ability to detect a PK-PD relationship in the alveolar macrophage.

AUC/MIC and *C*_max_/MIC for first-line drugs have been described as major drivers of treatment efficacy in preclinical models [36–39], but local and regional variation in MICs of non-genotypically resistant *Mtb* isolates against first-line TB drugs have not been well described. Baseline isolates in Malawi were found to be remarkably sensitive to rifampicin. Although we might expect higher MIC values to be associated with poorer outcomes [40], we observed little meaningful variability in MIC in this cohort. High plasma and intrapulmonary AUC/MIC and *C*_max_/MIC ratios are achieved in these patients as a result of preserved drug sensitivity, particularly for rifampicin. The significant association between ELF RIF and INH levels and bacillary clearance was also lost when we factored in MIC and may reflect the small number of cases with complete intrapulmonary PK and MIC data.

Taken together, these data suggest that favorable intrapulmonary tissue penetration is an important determinant of the sterilizing activity of a regimen. Reappraisal of drug exposure-response relationships for first-line TB treatment will enable optimization of treatment. Dose refinement, even using the existing drugs in our armamentarium, offers hope that transmission reduction and treatment abbreviation are possible.

## Supplementary Data

Supplementary materials are available at *Clinical Infectious Diseases* online. Consisting of data provided by the authors to benefit the reader, the posted materials are not copyedited and are the sole responsibility of the authors, so questions or comments should be addressed to the corresponding author.

ciac228_suppl_Supplementary_Material

## Notes

***Acknowledgments.*** The authors thank the staff of the Clinical Investigation Unit, the TB ward at Queen Elizabeth Central Hospital, the TB Officers of Blantyre District Health Office, and the Malawi-Liverpool-Wellcome Clinical Research Programme for their support and cooperation during the study. Lisa Stone was integral to some of the laboratory work described in this article but sadly passed away before it was completed; they gratefully acknowledge her contribution. They are grateful to Elizabeth Joekes for her advice on the chest radiograph scoring. Finally, they would like to thank all study participants for their enthusiastic participation in the study, without whom this work would not be possible.

***Financial support.*** This work was supported by a Wellcome Trust Clinical PhD Fellowship (grant numbers 105392/B/14/Z to A. D. M. and L69AGB to J. M.). E. L. C. was supported by Wellcome (grant number 200901/Z/16/Z). The Malawi-Liverpool-Wellcome (MLW) Clinical Research Programme is supported by a strategic award from the Wellcome Trust (award number 206545/Z/17/Z). They also acknowledge infrastructural support for bioanalysis from the Liverpool Biomedical Research Centre funded by Liverpool Health Partners.
