# Supplementary material for: High Intrapulmonary Rifampicin and Isoniazid Concentrations Are Associated With Rapid Sputum Bacillary Clearance in Patients With Pulmonary Tuberculosis
Source: Clin Infect Dis. 2022 Mar 23;75(9):1520–8. doi: 10.1093/cid/ciac228 (PMC9617580; doi:10.1093/cid/ciac228)
Supplement: ciac228_suppl_Supplementary_Material [file ciac228_suppl_supplementary_material.docx]

**Supplementary Materials**

**Supplementary Table 1: Parameter estimates for bacillary elimination rate model**

| **Fixed effects, log_10_(1/TTP) ~ days on treatment** | | | |
| --- | --- | --- | --- |
| **Parameter** | **Estimate** | **SE** | **RSE (%)** |
| Intercept (a) | -0.979 | 0.008 | 8.0 |
| CXR | 0.013 | 0.007 | 54.6 |
| Slope (b) | -0.016 | 0.001 | 7.4 |
| Sigma | -0.293 | 0.048 | 9.4 |
| **Random effects, ~ 1 + days \| participant** | | | |
| **Parameter** | **Estimate** | **SE** | **RSE (%)** |
| Intercept + CXR (a + CXR/33.4) | 0.104 | 0.026 | 24.6 |
| Slope (b) | 0.288 | 0.096 | 33.4 |

Model fit by maximum likelihood with observations below the limit of detection treated as censored. Sputum time-to-positivity (TTP) results have been transformed into the log of their reciprocal. The intercept (a) represents the baseline bacillary load, and the slope (b) the bacillary elimination rate (BER). Baseline chest radiograph score is included as CXR. Sigma represents the parameterised standard deviation on the residual error.

SE: standard error; RSE: residual standard error.

**Supplementary Figure Legends**

**Supplementary Figure 1: CONSORT diagram showing participant screening, recruitment, and follow-up**

New TB patients were identified by Blantyre TB Officers and referred to the study team for screening and enrolment. Patients remained in the study over the course of TB treatment and were followed up for 12 months post-treatment. Abbreviations: LTFU: lost to follow-up.

^a^ Patients were excluded post-recruitment if none of the sputum samples were positive for *Mycobacterium tuberculosis* on culture or Xpert MTB/RIF. Participants were allocated to the Intrapulmonary or Plasma Arms sequentially.

^b^ Death was classified as ‘definitely or probably attributable to active TB’ or ‘attributable to causes other than active TB’ after consultation between the study clinician and a physician from Queen Elizabeth Central Hospital independent from the study team. Only deaths attributable to TB were included as unfavourable outcomes. Two participants died during TB re-treatment and were classed as unfavourable outcomes.

^c^ Participants with negative tuberculosis sputum cultures from end-of-treatment onwards or who stopped coughing and remained well after treatment were defined as having a favourable clinical outcome.

^d^ Participants that were culture-negative at end-of-treatment and were subsequently diagnosed with recurrent TB (clinically, radiologically, or microbiologically) were considered to have relapsed. Those with positive cultures at end-of-treatment were deemed to have failed treatment. Recurrent TB, failed treatment, and death due to TB were grouped together as unfavourable clinical outcomes.

**Supplementary Figure 1: CONSORT diagram showing participant screening, recruitment, and follow-up**

**
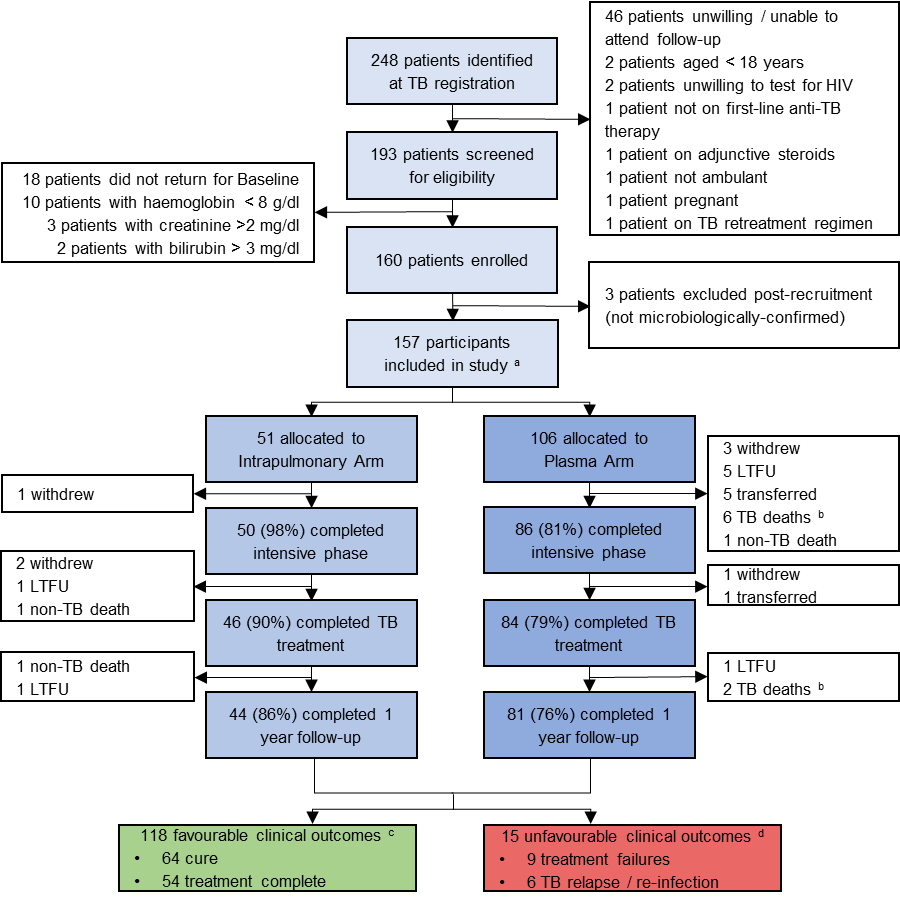
**
